# Socioeconomic Inequalities in Health Service Utilization in the Peruvian Population: A National Population-Based Study

**DOI:** 10.3390/healthcare14152238

**Published:** 2026-07-23

**Authors:** Miguel A. Arce-Huamani, Dick Hendric Castañeda-Aguilar

**Affiliations:** Escuela de Posgrado, Universidad Continental, Lima 15046, Peru; dcastaneda@continental.edu.pe

**Keywords:** socioeconomic characteristic, health service utilization, health equity, healthcare utilization, poverty

## Abstract

**Background/Objectives**: Socioeconomic inequalities continue to shape health service use in low- and middle-income countries. This study aimed to quantify socioeconomic inequalities in formal health service utilization among Peruvian adults using nationally representative data. **Methods**: A cross-sectional analytical study was conducted using the 2023 National Household Survey (ENAHO). The sample included 77,793 adults aged 18 years or older. Formal health service utilization was defined as self-reported use of outpatient, emergency, or hospitalization services during the preceding 12 months. Survey-weighted Poisson regression with robust variance was used to estimate prevalence ratios (PRs) and 95% confidence intervals (CIs). **Results**: Overall, 15.94% of adults reported using formal health services in the previous year. In the adjusted model, utilization was higher among women (aPR: 1.47; 95% CI: 1.41–1.55) and adults aged 60 years or older (aPR: 1.74; 95% CI: 1.57–1.92). Compared with uninsured adults, service utilization was higher among those covered by SIS (aPR: 2.59; 95% CI: 2.29–2.93), EsSalud (aPR: 2.90; 95% CI: 2.56–3.29), Armed Forces/Police insurance (aPR: 3.04; 95% CI: 2.36–3.91), and private insurance (aPR: 2.32; 95% CI: 1.77–3.03). Non-extreme poverty (aPR: 0.72; 95% CI: 0.67–0.78) and extreme poverty (aPR: 0.68; 95% CI: 0.59–0.78) were associated with lower utilization. **Conclusions**: Formal health service utilization remains low and unequal in Peru. Insurance coverage was strongly associated with greater service use, whereas poverty was associated with lower utilization.

## 1. Introduction

Socioeconomic inequalities in health service utilization continue to be a central challenge in global health [1,2]. Evidence from different settings shows that poverty, low educational attainment, lack of insurance, rural residence, and geographic barriers remain important determinants of unequal healthcare use. In sub-Saharan Africa, socioeconomic disadvantage has been associated with barriers to healthcare among married women [3]. In China, national surveys have described persistent income-related inequalities in perceived access to health services among older adults [4]. Similar inequities have also been reported among vulnerable populations in Spain and other settings [5,6], in maternal healthcare utilization in Togo [7], among female youths in Mozambique [8], and in timely emergency care in the United Kingdom [9]. These findings show that socioeconomic inequalities in healthcare use are a global concern, but their magnitude and determinants depend strongly on each country’s health-system structure and social context.

In Peru, socioeconomic inequalities in health service utilization must be interpreted within a segmented and fragmented health system. Healthcare is provided mainly through the Ministry of Health and regional government facilities, EsSalud, the Armed Forces and Police health services, and the private sector. The Ministry of Health and regional facilities serve a large share of the population, particularly people covered by the Comprehensive Health Insurance scheme (Seguro Integral de Salud, SIS), which is intended to protect poor, vulnerable, and uninsured populations. EsSalud covers formal-sector workers and their dependents, whereas the Armed Forces and Police have separate provider networks. Private insurance and out-of-pocket payment coexist with these public arrangements, especially among higher-income groups [10,11].

Healthcare services are organized across primary care facilities, secondary-level hospitals, and tertiary referral hospitals. However, the distribution of infrastructure, health personnel, medicines, and specialized services remains uneven across the country, particularly between urban areas and rural highland or jungle regions [10,11]. Primary care facilities are usually the first point of contact, especially for SIS affiliates, but access to specialized or hospital-based care often depends on referral pathways, appointment availability, transportation, and the capacity of local facilities. Therefore, insurance coverage does not necessarily guarantee timely or effective service use. In this context, socioeconomic position, insurance status, and area of residence may operate as key determinants of formal health service utilization in Peru.

Although socioeconomic gradients in health service utilization have been widely documented in low- and middle-income countries, including Latin America [12], updated national evidence for Peru remains necessary. Previous studies using the National Household Survey (Encuesta Nacional de Hogares, ENAHO) have documented pro-rich patterns in medical consultation use and highlighted the contribution of economic position, insurance coverage, and place of residence to these inequalities [13]. However, updated estimates using ENAHO 2023 are still needed to characterize current socioeconomic gradients in formal health service utilization and to inform equity-oriented policy decisions in the post-pandemic period.

In Peru, socioeconomic inequality also intersects with pronounced geographic and ethnolinguistic heterogeneity. For this reason, poverty status and educational attainment were treated as core indicators of socioeconomic position, while health insurance and area of residence captured enabling and structural constraints. Mother tongue was also included because first language is a relevant marker of ethnolinguistic marginalization and may reflect communication barriers and discriminatory experiences that shape health-seeking behavior and service use [14]. Sex, age, and marital status were incorporated as predisposing sociodemographic characteristics to reduce confounding when estimating socioeconomic gradients in utilization.

For conceptual clarity, this study distinguishes between health service utilization, access to healthcare, and unmet need. Health service utilization refers to the actual use of formal healthcare services, such as outpatient care, emergency care, or hospitalization. Access to healthcare is a broader concept that includes the opportunity to obtain timely, affordable, acceptable, and appropriate care when needed. Unmet need refers to situations in which individuals require healthcare but do not receive it. Because ENAHO 2023 records reported service use but does not fully capture clinical need, perceived need, or all barriers faced by those who did not seek care, the present study focuses on formal health service utilization. Therefore, the findings are interpreted as inequalities in realized service use, rather than as direct measures of access barriers or unmet healthcare need.

Therefore, the objective of this study was to quantify socioeconomic inequalities in formal health service utilization among Peruvian adults using nationally representative ENAHO 2023 data.

## 2. Materials and Methods

### 2.1. Study Design and Data Source

We conducted a quantitative, observational, analytical, cross-sectional study using secondary data from the 2023 National Household Survey (ENAHO), conducted by the National Institute of Statistics and Informatics (INEI) of Peru. ENAHO is a nationally representative, population-based survey designed to monitor socioeconomic, demographic, and health-related indicators in the Peruvian population.

Data collection was conducted from January to December 2023 through structured, face-to-face interviews using standardized questionnaires. The sampling frame was based on the National Household Register and followed a probabilistic, stratified, multistage sampling design that included urban and rural areas across all regions of Peru.

This study was designed to evaluate socioeconomic inequalities in formal health service utilization among Peruvian adults. Because the outcome reflects realized use of services, the findings were interpreted as inequalities in health service utilization, considered a proxy for realized access, rather than as a direct measure of access barriers or unmet need.

### 2.2. Study Population, Sample Size, and Sampling

The source population consisted of all household members included in ENAHO 2023. For this secondary analysis, the target population was restricted to adults aged 18 years or older living in private households. The initial ENAHO 2023 dataset included 119,747 household members. After restricting the dataset to adults aged 18 years or older, the eligible adult sample included 77,793 adults. Missing data for variables required in the adjusted models were minimal and were limited to educational level and mother tongue. Specifically, 143 adults had missing information on educational level, and 60 adults had missing information on mother tongue; the 60 records with missing mother tongue were included among the 143 records with missing educational level. Therefore, the complete-case sample used for adjusted regression analyses included 77,650 adults.

The selection of the analytical sample was based exclusively on age, residence in private households, and availability of complete information for the study variables. No participant was selected or excluded according to health service utilization status or according to the direction or magnitude of the associations. Therefore, the final sample corresponds to the eligible adult population with analyzable ENAHO 2023 data.

No additional sample size calculation was performed because this was a secondary analysis of an existing nationally representative survey. The final sample size was determined by the number of eligible adults with valid information after applying the inclusion and exclusion criteria. All analyses incorporated the complex survey design and sampling weights provided by INEI to obtain nationally representative estimates.

### 2.3. Eligibility Criteria

Participants were eligible if they were adults aged 18 years or older, lived in private households, and were included in the ENAHO 2023 individual and household-level datasets. Participants were also required to have complete information on formal health service utilization and all sociodemographic, socioeconomic, insurance-related, and geographic variables included in the analysis.

Participants were excluded if they had missing data for any study variable. Individuals without complete information required to construct the outcome, exposure variables, or covariates were not included in the final analytical sample.

### 2.4. Variables and Definitions

The primary outcome was formal health service utilization, defined as self-reported use of any formal health service during the preceding 12 months. This binary variable was coded as yes or no and was constructed from ENAHO items capturing outpatient care, emergency care, or hospitalization service use.

Because this measure represents realized service use, it may reflect both the ability to obtain care and differences in underlying health needs. Therefore, the results were interpreted as socioeconomic inequalities in health service utilization, rather than as a direct measure of unmet need or structural access barriers.

The main independent variables included sex, age group, educational level, marital status, mother tongue, type of health insurance, area of residence, and poverty status. Sex was categorized as male or female. Age was grouped into 18–29, 30–39, 40–59, and 60 years or older. Educational level was categorized as no education, primary, secondary, or higher education. Marital status was categorized as single, cohabiting, married, widowed, or divorced/separated. Mother tongue was categorized as Spanish or Quechua, Aymara, or other language.

Type of health insurance was categorized as uninsured, Seguro Integral de Salud (SIS), EsSalud, Armed Forces or Police health insurance, and private or other insurance. Area of residence was categorized as urban coast, rural coast, urban highlands, rural highlands, urban jungle, rural jungle, and Metropolitan Lima/Callao. Poverty status was categorized as non-poor, non-extreme poor, or extreme poor, according to the ENAHO poverty classification.

Covariates were selected a priori using Andersen’s Behavioral Model of Health Service Use, adapted to the Peruvian context [15,16]. According to this framework, health service utilization is shaped by predisposing factors, enabling resources, and need-related factors. Sex, age group, marital status, and mother tongue were considered predisposing characteristics. Educational attainment and poverty status were treated as socioeconomic indicators that shape the social position of individuals and households. Health insurance and area of residence were considered enabling factors because they influence the ability to obtain and navigate formal healthcare services. Although underlying health need is a central component of Andersen’s model, ENAHO 2023 does not provide sufficiently detailed clinical information to adjust for morbidity or severity of health conditions in this analysis. Therefore, the results should be interpreted as inequalities in realized service use after adjustment for available sociodemographic, socioeconomic, insurance-related, and geographic factors, rather than as fully need-standardized inequalities.

Among individuals who reported formal health service utilization, out-of-pocket health expenditure was estimated by dividing total household health expenditure by the number of adult household members who reported service utilization. The resulting value was converted from Peruvian soles to US dollars using an exchange rate of 1 USD = 3.7 PEN. This variable was categorized into tertiles, corresponding to low, medium, and high out-of-pocket expenditure, and was used for descriptive analysis.

### 2.5. Data Management Procedures

Data management was performed using the ENAHO 2023 individual- and household-level datasets. The analytical process included merging the required ENAHO modules, identifying eligible adults, recoding variables according to the ENAHO codebook and technical documentation, and constructing the outcome and covariates according to the predefined analytical workflow.

The final database was reviewed for consistency, missing values, and coding errors before statistical analysis. All analyses used anonymized public-use microdata. No direct contact with participants was performed, and no intervention was implemented as part of this secondary analysis. Data management and statistical analyses were performed using Stata version 18 (StataCorp, College Station, TX, USA). All analyses incorporated the complex survey design of ENAHO using the INEI-provided sampling weights, stratification variables, and primary sampling units. The sampling weight factor07 was applied to obtain nationally representative estimates.

Descriptive statistics were used to summarize the distribution of the study variables. Categorical variables were presented as weighted proportions and corresponding frequencies. Bivariate associations between each covariate and formal health service utilization were evaluated using the Rao–Scott chi-square test for survey data.

Poisson regression models with robust sandwich variance estimators were used to estimate prevalence ratios (PRs) and 95% confidence intervals (CIs) for the association between sociodemographic, socioeconomic, insurance-related, and geographic factors and formal health service utilization. This modeling strategy was selected because the outcome was binary and not rare, allowing direct estimation of prevalence ratios rather than odds ratios.

The final multivariable model included sex, age group, educational level, marital status, mother tongue, type of health insurance, area of residence, and poverty status. All regression analyses accounted for the complex survey design, including sampling weights, strata, and primary sampling units. Statistical significance was set at *p* < 0.05.

Missing data were assessed before model fitting. Descriptive analyses reported available data for each variable, whereas regression models used complete-case analysis. Because missingness was low and limited to educational level and mother tongue, only 143 adults were excluded from the adjusted regression models due to missing covariate information.

To further quantify socioeconomic inequality in formal health service utilization, we estimated the standard concentration index. Adults were ranked according to per capita household total expenditure, calculated as total household expenditure divided by the number of household members. The concentration index ranges from −1 to +1, where a positive value indicates that service utilization is concentrated among individuals with higher socioeconomic position, a negative value indicates concentration among individuals with lower socioeconomic position, and zero indicates no socioeconomic-related inequality. The analysis incorporated the ENAHO sampling weights, and 95% confidence intervals were estimated using cluster-stratified bootstrap procedures.

Exploratory stratified analyses were conducted to assess whether the association between poverty status and formal health service utilization was consistent across key population groups. Stratified models were fitted by sex, age group, and health insurance status. For health insurance, categories were collapsed into insured versus uninsured to avoid unstable estimates in small insurance subgroups. Within each stratum, poverty status was the main socioeconomic exposure, and models were adjusted for the remaining covariates not used as the stratification variable. These analyses were interpreted as exploratory.

### 2.6. Ethical Considerations

The original research protocol for this study, based on the same dataset as the doctoral thesis project entitled “Factores asociados con el acceso a los servicios de salud en la población peruana, 2023,” was approved by the Institutional Research Ethics Committee of Universidad Continental under Approval No. 1333-2024-CIEI-UC, issued in January 2025.

ENAHO 2023 obtained informed consent from all survey participants during primary data collection. The dataset used in this study is fully anonymized and publicly available through the INEI microdata repository, accessed on 11 June 2025. Therefore, no additional informed consent was required for this secondary analysis.

All procedures were conducted in accordance with the ethical principles of the Declaration of Helsinki and applicable regulations for secondary analysis of anonymized public-use data.

## 3. Results

**Figure 1:**
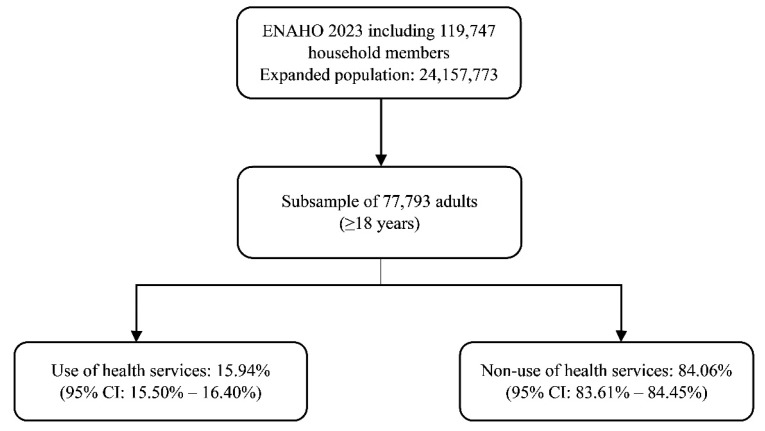
Flow diagram of sample selection and classification according to health service utilization, 2023.

As shown in Figure 1, ENAHO 2023 included 119,747 household members, corresponding to an expanded population of 24,157,773 individuals. After restricting the dataset to adults aged 18 years or older and excluding records without valid information for the outcome or covariates required for the analysis, the final analytical sample included 77,793 adults. This selection was based only on prespecified eligibility and data availability criteria and was not conditioned on health service utilization status. In this analytical sample, 15.94% (95% CI: 15.50–16.40%) reported using formal health services during the previous year, whereas 84.06% (95% CI: 83.61–84.45%) did not report using these services.

Missing data were uncommon in the eligible adult sample. Among 77,793 adults, 143 records had missing information on educational level, and 60 records had missing information on mother tongue. All records with missing mother tongue were also missing educational level. Therefore, 77,650 adults were included in the complete-case regression analyses. No missing data were observed for sex, age group, marital status, health insurance, area of residence, poverty status, or formal health service utilization.

Table 1 summarizes the weighted sociodemographic, socioeconomic, insurance-related, and geographic characteristics of the analytical sample. Overall, the study population included slightly more women than men, a broad distribution of adult age groups, and a predominance of adults with secondary or higher education. Most participants reported Spanish as their mother tongue and had some form of health insurance, mainly SIS or EsSalud. The sample also showed marked territorial heterogeneity, including urban coastal areas, Metropolitan Lima/Callao, highland areas, and jungle regions. Full weighted distributions are shown in Table 1.

**Figure 2:**
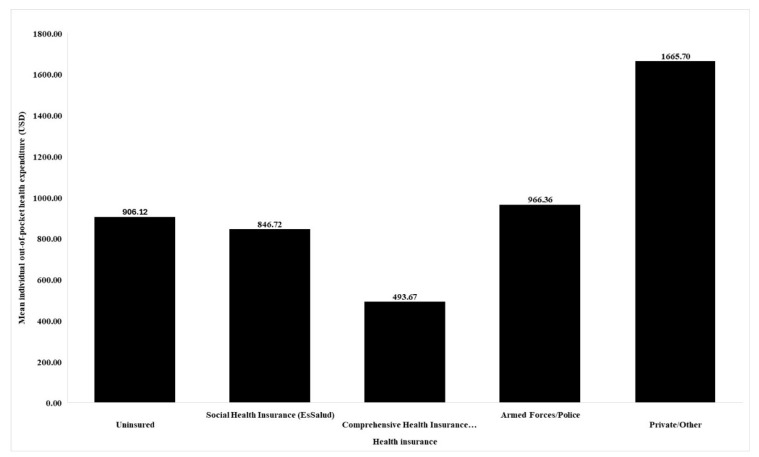
Mean individual out-of-pocket health expenditure by type of insurance, 2023.

Figure 2 displays the distribution of mean individual out-of-pocket health expenditures according to type of insurance. The highest expenditure was observed among those with private or other types of insurance (US$1665.70), followed by members of the Armed Forces/Police (US$966.36) and the uninsured (US$906.12). Affiliates of EsSalud also reported considerable expenditure (US$846.72), while those covered by Comprehensive Health Insurance (SIS) had the lowest mean expenditure (US$493.67).

**Figure 3:**
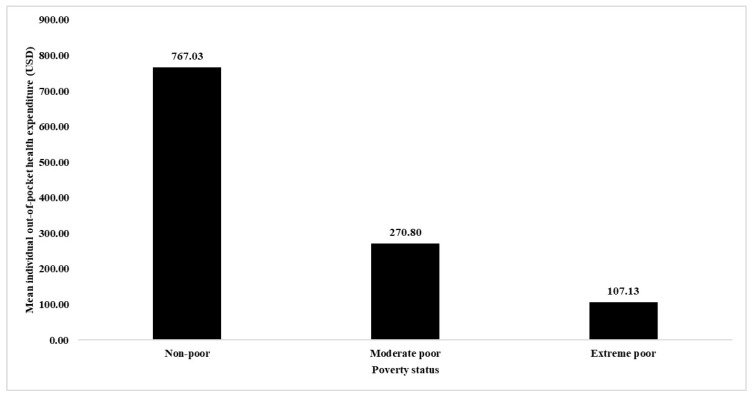
Out-of-pocket health expenditure according to poverty status, 2023.

As shown in Figure 3, out-of-pocket health expenditure varied sharply by poverty status. Non-poor individuals had the highest average expenditures (US$767.03), compared to moderate poor (US$270.80) and extremely poor (US$107.13) groups.

Table 2 presents the bivariate associations between study characteristics and formal health service utilization. Statistically significant differences were observed across the evaluated variables. In general, service utilization was more frequent among women, older adults, insured individuals, and non-poor adults. Differences were also observed according to educational level, marital status, mother tongue, area of residence, and poverty status. Because the full distribution by utilization status is provided in Table 2, the text highlights only the main patterns.

Table 3 shows the crude and adjusted prevalence ratios for formal health service utilization. In the multivariable model, women and older adults were more likely to use formal health services, with the strongest age-related association observed among adults aged 60 years or older. Health insurance was the main enabling factor associated with utilization, as all insurance categories showed substantially higher utilization compared with being uninsured. In contrast, poverty remained inversely associated with service use after adjustment, with both non-extreme poor and extreme poor adults showing lower utilization than non-poor adults. Geographic differences also persisted, particularly for rural highland, rural jungle, and urban jungle areas. Mother tongue was not independently associated with utilization after adjustment.

To quantify the magnitude and direction of socioeconomic-related inequality, we estimated the concentration index for formal health service utilization using per capita household total expenditure as the ranking variable. The concentration index was positive and statistically significant (C = 0.097; 95% CI: 0.083–0.111; *p* < 0.001), indicating that formal health service utilization was concentrated among adults with higher socioeconomic position (Table 4).

Exploratory stratified analyses showed that the inverse association between poverty status and formal health service utilization was generally consistent across sex, age group, and health insurance status (Table 5). Among both men and women, non-extreme and extreme poverty were associated with lower utilization compared with being non-poor. Similar patterns were observed across all age groups. When stratified by insurance status, the poverty gradient was particularly marked among uninsured adults, although it also persisted among insured adults.

## 4. Discussion

This study aimed to identify socioeconomic inequalities in formal health service utilization in the Peruvian adult population using nationally representative ENAHO 2023 data. The findings show that formal health service utilization remained low, with only 15.94% of adults reporting use during the previous year, and that service use was patterned by socioeconomic, insurance-related, and territorial factors. Health insurance was the strongest enabling factor associated with utilization, whereas poverty was consistently associated with lower service use after adjustment. These results indicate that, despite progress toward broader health coverage, important inequalities in realized use of formal health services persist in Peru. Therefore, equity-oriented policies should go beyond insurance expansion and address the structural barriers faced by poor, uninsured, and geographically disadvantaged populations.

An important consideration is that formal health service utilization reflects realized service use and may be influenced by both access-related factors and underlying health need. Higher utilization among some groups, such as older adults or residents of rural highland and jungle areas, may partly reflect greater morbidity, greater perceived need for care, or more frequent contact with the health system because of chronic or acute conditions. Conversely, lower utilization among poor, uninsured, or geographically disadvantaged adults may indicate access barriers, delayed care-seeking, lower perceived need, or unmet healthcare needs that were not directly measured in ENAHO 2023. Therefore, the results should be interpreted as socioeconomic inequalities in realized utilization rather than as definitive evidence of equal or unequal healthcare need.

These findings are consistent with the social determinants of health framework proposed by the WHO Commission on Social Determinants of Health, which emphasizes that health inequities are produced by the unequal distribution of power, income, resources, and opportunities. In the Peruvian context, this mechanism is visible in the lower utilization observed among poor and extremely poor adults, as well as in the persistent differences by insurance status and place of residence. Geography is also central to this interpretation. Populations living in rural highland and jungle areas may face longer travel times, higher transportation costs, limited health workforce availability, and weaker referral capacity, all of which can restrict timely use of formal health services. Therefore, the results should not be interpreted only as individual differences in care-seeking behavior, but as evidence of structural barriers that shape realized access to healthcare in Peru [17,18,19,20].

The concentration index analysis strengthens this interpretation by providing a summary measure of socioeconomic-related inequality. The positive concentration index indicates that formal health service utilization was disproportionately concentrated among adults with higher per capita household expenditure. This finding is consistent with the adjusted regression results, in which poverty remained inversely associated with service utilization, and with previous Peruvian evidence describing pro-rich patterns in medical consultation use. Therefore, the inequality observed in this study is not only reflected in group-specific prevalence ratios, but also in a measurable socioeconomic gradient in the distribution of realized service use.

Beyond statistical significance, the practical meaning of these findings should be emphasized. The overall prevalence of formal health service utilization was only 15.94%, indicating that most adults did not report using outpatient, emergency, or hospital services during the previous year. The magnitude of the adjusted associations was also relevant from a public health perspective. Insurance coverage was associated with more than a twofold higher prevalence of service utilization compared with being uninsured, while poverty was associated with approximately 28–32% lower utilization after adjustment. In addition, the positive concentration index confirmed that service use was disproportionately concentrated among adults with higher socioeconomic position. Taken together, these results suggest that the observed inequalities are not only statistically detectable but also meaningful for health-system planning, particularly in relation to insurance effectiveness, poverty reduction, territorial service availability, and financial protection.

From a Peruvian health-system perspective, the main contribution of this study is that it shows how socioeconomic inequality in formal service utilization persists within a segmented system in which insurance affiliation, territorial capacity, and household economic position continue to shape realized service use. These findings are consistent with previous ENAHO-based evidence documenting pro-rich inequality in medical consultation use and showing the contribution of economic position, insurance coverage, and place of residence to observed gaps [13]. They also align with evidence that health-system segmentation and fragmentation can generate unequal opportunities to obtain timely care across socioeconomic and territorial groups in Peru [10,11]. Therefore, although international comparisons are useful for contextualizing the findings, the interpretation of this study should remain centered on Peru’s institutional structure, the role of SIS and EsSalud, persistent poverty-related barriers, and the unequal availability of services across coastal, highland, jungle, and Metropolitan Lima/Callao settings.

In this study, women were more likely than men to use formal health services. In São Paulo, older women experienced more difficulty using health services than men, 42.2% compared to 30.9% [21]. In China, health inequalities were more severe among women, especially in rural areas [22]. In contrast, the South African adult population showed a nearly equal distribution of women and men utilizing health services, 52% and 48% respectively [23]. Similarly, the gender distribution in health service utilization in Peru remained stable over time, with 51% women and 49% men, showing no significant difference [24]. These patterns likely reflect differences in gender roles, social norms, and health-seeking behaviors across countries, while in some settings, economic or systemic barriers may outweigh the impact of gender itself. Advancing gender equity in healthcare utilization demands context-sensitive strategies that address both social determinants and the structures shaping health system responsiveness for women and men.

Older adults exhibited a greater likelihood of utilizing health services compared to younger groups. In São Paulo, 41.1% of people aged 60–69 had trouble using health services, while this percentage declined to 35.2% for those aged 70–79 and 28.1% for those aged 80 or above [21]. Studies from Mozambique found that female youths aged 20–24 had 15% lower odds of facing barriers to healthcare utilization than those aged 15–19 (AOR = 0.85; 95% CI: 0.73, 0.99) [8]. In South Africa, age made a statistically significant but modest contribution to inequality in private sector healthcare utilization, accounting for only 1.96% of the explained variance [23]. The wealth–health gradient becomes more pronounced as people age; greater wealth is associated with better health among older adults, while lower economic status leads to poorer health and increased disability [25]. These findings may reflect a combination of increased health needs in older populations and differences in health-seeking behavior across the life course but also highlight the ongoing presence of financial, geographic, and cultural barriers among younger individuals. Promoting equitable health service utilization requires interventions that consider the specific barriers encountered by both older and younger adults, ensuring no age group is left behind.

Individuals with primary education showed a slightly higher adjusted prevalence of formal health service utilization than those with no formal education. This finding should be interpreted cautiously. In the Peruvian context, having at least primary education may improve basic health literacy, communication with providers, understanding of administrative procedures, and navigation of public services compared with having no formal schooling. However, the absence of a clear gradient across secondary and higher education suggests that education alone does not explain service use once insurance status, poverty, residence, age, and other sociodemographic factors are considered.

Evidence from other settings supports the relevance of education, although its relationship with service utilization varies across health-system contexts. In the United States, 49.9% of uninsured Latina mothers had less than a high school education, while only 12.2% of mothers with private insurance fell into this category [26]. In Mozambique, youths with no formal education, primary education, and secondary education had higher odds of facing barriers to care compared with those with higher education [8]. Brazilian data showed that health service utilization, professional contact, and preventive care were more prevalent among individuals with higher education [27]. In Ecuador, socioeconomic inequalities in skilled birth attendance by education decreased significantly over time, while disparities in cervical cancer screening by education increased [28]. In São Paulo, absolute and relative inequalities by education decreased between 2006 and 2015, with only 20.7% of those with 12 or more years of education reporting difficulty accessing health services, compared with 44.2% among those with less than one year [21]. In China, higher educational attainment has been consistently associated with better health and lower health inequality among older adults [22]. Taken together, these findings suggest that education can facilitate health-system navigation and care-seeking, but its effect on realized service utilization depends on the broader health-system context. In Peru, insurance coverage and territorial availability of services may be more immediate determinants of formal service utilization than educational attainment alone.

Married, cohabiting, widowed, and divorced or separated adults experienced greater health service utilization relative to their single counterparts. In the United States, married mothers were more likely to have private insurance for their children, with a coverage rate of 75.7%, while children of mothers who were never married faced higher rates of uninsurance at 25.7% [26]. In Mozambique, married female youths had a 1.22 times higher likelihood of facing barriers to healthcare utilization compared to single youths (AOR = 1.22; 95% CI: 1.03, 1.44) [8]. These contrasting results may reflect differences in how marital status influences social support, economic stability, and eligibility for health coverage across settings. In some contexts, being married may provide increased utilization through family insurance or greater household resources, while in others, it may introduce additional barriers, particularly for young women.

Having any form of health insurance was strongly associated with increased health service utilization. In the United States, youth with uninsured mothers were substantially more likely to be uninsured themselves, with 18.9% lacking coverage compared to only 2.2% among those whose mothers had private insurance; maternal uninsurance was powerfully linked to youth uninsurance (OR = 23.2, 95% CI: 17.2–31.1) [26]. In South Africa, households at an economic advantage were more likely to have private medical insurance, reflected by a concentration index of +0.490 (*p* < 0.001) [23]. Expanding health insurance coverage in the United States, through initiatives such as state Medicaid expansions, resulted in decreased rates of uninsured older adults, fewer people forgoing care due to costs, and lower mortality [25]. In Peru, national coverage increased from 63.2% in 2010 to 75.5% in 2016, suggesting gradual improvements in population protection [24]. In China, increased utilizationibility to medical services weakened the correlation between socioeconomic deprivation and health inequality, underscoring the protective effect of health insurance and service coverage [22]. These consistent findings highlight the central role of insurance in facilitating entry into the health system and reducing financial barriers, while also revealing persistent gaps for the uninsured or underinsured. Achieving universal health coverage remains a foundational step for closing inequities in health service utilization and realizing the promise of equitable healthcare for all.

Living in rural highland and rural jungle areas showed a significant association with higher formal health service utilization, while urban jungle areas reflected a different pattern. This finding should be interpreted cautiously and should not be read as evidence that rural populations have better access to healthcare. In the Peruvian context, higher utilization in rural highland and Amazonian populations may reflect greater underlying health need, delayed care-seeking until illness becomes more severe, or reliance on public services when care is finally obtained, rather than timely or equitable access.

Among US-born Latino youth, those living in the Southern region of the United States had the highest odds of being uninsured, consistent with regional policy restrictions [26]. In South Africa, most respondents lived in urban areas, with notable disparities in healthcare utilization between urban and rural populations [23]. In Mozambique, female youths residing in rural areas had three times higher odds of facing barriers to healthcare utilization compared with urban residents (AOR = 3.00; 95% CI: 2.24, 4.01), and regional disparities were prominent [8]. In Ecuador, inequalities in skilled birth attendance by residence decreased significantly over time, yet disparities in screening coverage by residence remained unchanged [28]. In China, health inequalities were more severe in rural areas (RIF_CI = 0.10) than urban areas (RIF_CI = 0.09), and socioeconomic deprivation was greater in rural and central regions [22]. These differences reflect the combined effects of geographic barriers, resource allocation, health needs, and regional policy environments on realized service use. Bridging urban–rural and regional gaps will require targeted investments, improved infrastructure, stronger referral pathways, and context-sensitive interventions to ensure that all communities, regardless of geography, can benefit from accessible and equitable health services.

Individuals with lower socioeconomic status faced greater barriers to using health services. In Peru, only 12.9% of the poorest quintile had a medical consultation compared to 39.5% in the richest quintile [13]. Uninsured mothers in the United States were more likely to have household incomes below the federal poverty level, 43.3% versus 5.4% among mothers with private insurance [26]. In South Africa, the poor were disadvantaged across all dimensions of utilization, with healthcare needs and disability concentrated in the lowest-income groups, while the wealthiest reported a greater perceived desire for care [23]. In Brazil, the prevalence of medical consultations in the last year was 80.0%, but with marked inequalities by wealth; screening, vaccination, and contact with health professionals were also more common among the richest [27]. In Mozambique, youths from poor households had significantly higher odds of facing barriers to care (AOR = 1.9; 95% CI: 1.44, 2.49) [8]. In Ecuador, inequalities in skilled birth attendance and screening by household wealth decreased slightly, yet persistent gaps remained [28]. In China, socioeconomic deprivation was strongly associated with health inequality, and income emerged as the most important factor affecting health inequality among older adults [22]. These consistent findings across diverse contexts underscore how poverty and deprivation continue to restrict healthcare use and shape health inequities.

The exploratory stratified analyses further support the robustness of the poverty gradient. Lower utilization among poor and extremely poor adults was observed among both men and women, across all age groups, and among both insured and uninsured adults. The stronger poverty gradient among uninsured adults suggests that the combination of economic disadvantage and lack of coverage may create a particularly restrictive pathway to formal healthcare use. However, poverty also remained associated with lower utilization among insured adults, reinforcing the idea that insurance coverage alone is not sufficient to eliminate socioeconomic inequalities in realized service use.

The burden of out-of-pocket health by both type of insurance and poverty expenditures varied notably by both type of insurance and poverty status. This finding has important implications for financial protection. Higher average spending among non-poor adults and among those with private or other insurance should not be interpreted only as greater financial burden; it may also reflect greater capacity to pay, use of higher-cost services, or access to services outside the public sector. Conversely, lower spending among poor and extremely poor adults may reflect financial protection through SIS coverage, but it may also indicate deferred care, forgone services, limited access to medicines, or inability to pay for diagnostic tests, transportation, and specialist care. Therefore, out-of-pocket expenditure should be interpreted together with utilization patterns, poverty status, and insurance coverage.

Evidence from South Africa shows that economically disadvantaged households report greater difficulty paying for medical care and medicines, even when the absolute level of spending is lower than that of wealthier groups [23]. Similarly, in the United States, cost has been identified as a major reason for not receiving needed care among older adults [25]. In Peru, reducing out-of-pocket spending should remain a central component of universal health coverage, particularly for medicines, diagnostic procedures, transportation, and referral-related costs. Strengthening medicine availability in public facilities, improving coverage of diagnostic services, reducing informal or administrative costs, and supporting transportation for rural and remote populations may help prevent financial barriers from becoming barriers to realized service use.

The policy implications of these findings are direct. First, Peru should strengthen the first level of care in poor, rural highland, and jungle areas by improving staffing, medicine availability, diagnostic capacity, referral systems, and transportation support. Second, insurance expansion should be accompanied by effective access guarantees, because formal coverage alone does not ensure timely outpatient, emergency, or hospital care. Third, SIS-affiliated and uninsured populations require simplified administrative pathways, active enrollment strategies, and better continuity of care between primary care facilities and referral hospitals. Fourth, territorial planning should prioritize areas where poverty, geographic isolation, and weak service supply overlap, particularly rural Andean and Amazonian territories. Finally, monitoring health service utilization with ENAHO and other national data sources should become part of routine equity surveillance, allowing policymakers to identify whether public investments are actually reducing unjust differences in realized access. These actions would move the response beyond broad universal health coverage goals toward measurable, equity-oriented implementation.

Peru has current national planning instruments that can support this equity-oriented agenda. The National Multisectoral Health Policy to 2030, “Perú, País Saludable,” provides a long-term framework based on primary healthcare, territorial management, social determinants of health, equity, and life-course care [29]. In addition, the Health Sector Multiyear Strategic Plan 2024–2030 establishes sectoral priorities and planning lines for health-system development [30]. However, the challenge is not only the existence of policy documents, but their sustained implementation across political cycles. The present findings suggest that these national strategies should prioritize measurable reductions in unjust differences in service utilization, especially among poor, uninsured, rural highland, and Amazonian populations. This requires stable financing, territorial targets, strengthened primary care networks, improved referral capacity, and routine equity monitoring using nationally representative data.

This study offers several important strengths. The use of a large, nationally representative dataset ensures that the findings are generalizable to the adult population of Peru and provide robust statistical power to explore complex socioeconomic gradients in health service utilization. The inclusion of a wide range of sociodemographic variables and detailed subgroup analyses allows for a nuanced and comprehensive assessment of the patterns and determinants of inequality. The analytic approach—incorporating adjustment for the complex survey design and use of multivariable models—strengthens the validity and reliability of the results.

Nevertheless, some limitations should be considered. First, the cross-sectional design restricts the ability to draw causal conclusions from the associations observed. Reverse causality is also possible. Adults with greater health needs, especially older adults or individuals with chronic conditions, may be more likely to use formal health services because they require more frequent care. Therefore, higher utilization in some groups should not automatically be interpreted as better access or greater equity. Conversely, lower utilization among poor or uninsured adults may reflect barriers to care, lower perceived need, delayed care-seeking, or unmet need that could not be directly measured in ENAHO 2023. Second, all variables were based on self-report, which may introduce recall bias or social desirability bias, particularly for sensitive issues such as income, insurance status, or health service use. Third, the outcome captures healthcare utilization, or realized service use, and does not directly measure unmet need or barriers among individuals who required care but did not seek or obtain it; therefore, the findings should be interpreted as inequalities in utilization rather than definitive inequalities in access.

Certain subpopulations, including some Indigenous communities or those living in remote areas, may remain underrepresented despite the national scope. Additionally, residual confounding should be acknowledged. Although the analysis adjusted for key sociodemographic, socioeconomic, insurance-related, and geographic variables, ENAHO does not provide detailed clinical information on morbidity, disease severity, functional status, disability, chronic conditions, perceived health need, health literacy, quality of care, or local service supply. Unmeasured behavioral and social factors, such as health-seeking preferences, transportation availability, informal care networks, discrimination, and previous experiences with the health system, may also influence formal service utilization. Therefore, some observed associations may partly reflect unmeasured differences in underlying health need, service availability, or care-seeking behavior rather than socioeconomic position alone. Future research would benefit from longitudinal and mixed-methods designs to better capture the evolving and lived realities of health inequities in Peru.

## 5. Conclusions

This study successfully identified pronounced socioeconomic inequalities in health service utilization among the Peruvian adult population, based on comprehensive analysis of national survey data. The findings demonstrate that factors such as poverty, educational attainment, health insurance status, and area of residence remain key determinants of healthcare utilization. Despite recent policy advances and expanded insurance coverage, these structural barriers continue to prevent the most vulnerable groups, particularly the poorest, least educated, and rural residents, from fully benefiting from available health services. The evidence provided by this national study highlights not only the magnitude of existing disparities, but also the importance of prioritizing social determinants of health in future public health strategies.

## Figures and Tables

**Table 1:** Sociodemographic and socioeconomic characteristics of the Peruvian adult population, 2023.

Socioeconomic Characteristics	*n* (*N* = 77,793)	%
**Sex**		
Male	37,038	47.49%
Female	40,755	52.51%
**Age**		
18–29 years	18,282	24.20%
30–39 years	14,267	18.51%
40–59 years	26,647	34.32%
60 years and older	18,597	22.97%
**Educational level** ±		
No education	4286	4.87%
Primary	28,184	33.11%
Secondary	25,134	35.68%
Higher	20,046	26.34%
**Marital status**		
Single	19,272	27.15%
Cohabiting	22,117	27.60%
Married	21,509	26.65%
Widowed	5216	6.46%
Divorced/Separated	9679	12.14%
**Mother tongue** ±		
Spanish	59,802	79.11%
Quechua/Aymara/Other	17,931	20.89%
**Health insurance**		
Uninsured	9687	13.61%
SIS (Comprehensive Health Insurance)	48,025	58.24%
EsSalud (Social Health Insurance)	18,418	25.49%
Armed Forces/Police	892	1.35%
Private insurance	771	1.31%
**Area of residence**		
Urban Coast	21,366	21.15%
Rural Coast	3396	1.84%
Urban Highlands	15,022	20.26%
Rural Highlands	11,252	10.49%
Urban Jungle	10,111	7.87%
Rural Jungle	6081	3.35%
Metropolitan Lima and Callao	10,565	35.04%
**Poverty status**		
Non-poor	59,933	74.94%
Non-extreme poor	13,831	20.42%
Extreme poor	4029	4.64%
Out-of-pocket individual health expenditure (US$) *		659.35 (mean; 95% CI: 620.92–697.78)
Distribution by expenditure category *		
Low expenditure	3677	27.66%
Medium expenditure	4541	35.22%
High expenditure	4895	37.12%
**Health service utilization**		
No	64,680	84.06%
Yes	13,113	15.94%

Note: ± Variables with missing data: educational level had 143 missing values, and mother tongue had 60 missing values. The 60 records with missing mother tongue were included among the 143 records with missing educational level. Regression models used complete-case analysis and included 77,650 adults. * Measured only among those who reported formal health service utilization. SIS: Comprehensive Health Insurance; EsSalud: Social Health Insurance. Source: ENAHO 2023.

**Table 2:** Bivariate analysis of characteristics associated with formal health service utilization among Peruvian adults, 2023.

Socioeconomic Characteristics	Health Service Utilization				*p*-Value
	No		Yes		
	*n* (*N* = 64,680)	%	*n* (*N* = 13,113)	%	
**Sex**					
Male	32,196	49.58%	4842	36.49%	<0.001 *
Female	32,484	50.42%	8271	63.51%	
**Age**					
18–29 years	16,383	25.94%	1899	15.00%	<0.001 *
30–39 years	12,227	19.09%	2040	15.43%	
40–59 years	21,997	34.00%	4670	36.05%	
60 years and older	14,093	20.97%	4504	33.52%	
**Educational level**					
No education	3370	4.58%	916	6.33%	<0.001 *
Primary	22,657	31.87%	5527	39.68%	
Secondary	21,601	36.89%	3533	29.32%	
Higher	16,929	26.66%	3117	24.67%	
**Marital status**					
Single	17,194	28.89%	2078	17.94%	<0.001 *
Cohabiting	18,674	28.04%	3443	25.30%	
Married	17,192	25.73%	4317	31.49%	
Widowed	3855	5.76%	1361	10.18%	
Divorced/Separated	7765	11.58%	1914	15.09%	
**Mother tongue**					
Spanish	50,142	79.72%	9660	75.85%	<0.001 *
Quechua/Aymara/Other	14,480	20.28%	3451	24.15%	
**Health insurance**					
Uninsured	9143	15.26%	544	4.93%	
SIS	39,669	57.82%	8356	60.48%	
EsSalud	14,483	24.30%	3935	31.79%	
Armed Forces/Police	725	1.27%	167	1.75%	<0.001 *
Private insurance	660	1.35%	111	1.05%	
**Area of residence**					
Urban Coast	17,893	21.20%	3473	20.90%	0.003 *
Rural Coast	2841	1.85%	555	1.80%	
Urban Highlands	12,303	20.19%	2719	20.59%	
Rural Highlands	9152	10.28%	2100	11.61%	
Urban Jungle	8614	8.03%	1497	7.03%	
Rural Jungle	4968	3.28%	1113	3.75%	
Metropolitan Lima and Callao	8909	35.17%	1656	34.32%	
**Poverty status**					
Non-poor	49,186	73.80%	10,747	80.94%	<0.001 *
Non-extreme poor	11,975	21.35%	1856	15.51%	
Extreme poor	3519	4.85%	510	3.55%	

SIS: Comprehensive Health Insurance; EsSalud: Social Health Insurance. Statistically significant (* *p*-value < 0.05). Source: ENAHO 2023.

**Table 3:** Crude and adjusted prevalence ratios for formal health service utilization among Peruvian adults, 2023.

Socioeconomic Characteristics	Crude PR (95% CI)	*p*-Value	Adjusted PR (95% CI)	*p*-Value
**Sex**				
Male	Reference		Reference	
Female	1.57 (1.50–1.65)	<0.001	1.47 (1.41–1.55)	<0.001
**Age**				
18–29 years	Reference		Reference	
30–39 years	1.35 (1.24–1.46)	<0.001	1.15 (1.05–1.27)	0.003
40–59 years	1.69 (1.58–1.82)	<0.001	1.36 (1.24–1.49)	<0.001
60 years and older	2.35 (2.19–2.53)	<0.001	1.74 (1.57–1.92)	<0.001
**Educational level**				
No education	Reference		Reference	
Primary	0.92 (0.85–0.99)	0.046	1.14 (1.05–1.24)	0.002
Secondary	0.63 (0.58–0.69)	<0.001	0.97 (0.87–1.08)	0.561
Higher	0.72 (0.66–0.79)	<0.001	1.01 (0.91–1.13)	0.817
**Marital status**				
Single	Reference		Reference	
Cohabiting	1.39 (1.28–1.49)	<0.001	1.17 (1.07–1.28)	0.001
Married	1.79 (1.66–1.93)	<0.001	1.17 (1.08–1.29)	0.001
Widowed	2.38 (2.17–2.60)	<0.001	1.25 (1.11–1.39)	<0.001
Divorced/Separated	1.88 (1.72–2.06)	<0.001	1.32 (1.19–1.46)	<0.001
**Mother tongue**				
Spanish	Reference		Reference	
Quechua/Aymara/Other	1.21 (1.14–1.27)	<0.001	1.03 (0.97–1.10)	0.26
**Health insurance**				
Uninsured	Reference		Reference	
SIS	2.86 (2.54–3.24)	<0.001	2.59 (2.29–2.93)	<0.001
EsSalud	3.44 (3.04–3.89)	<0.001	2.90 (2.56–3.29)	<0.001
Armed Forces/Police	3.57 (2.77–4.60)	<0.001	3.04 (2.36–3.91)	<0.001
Private insurance	2.22 (1.69–2.89)	<0.001	2.32 (1.77–3.03)	<0.001
**Area of residence**				
Urban Coast	Reference		Reference	
Rural Coast	0.99 (0.87–1.12)	0.82	0.99 (0.88–1.12)	0.87
Urban Highlands	1.03 (0.96–1.10)	0.42	1.03 (0.96–1.11)	0.39
Rural Highlands	1.12 (1.04–1.20)	0.002	1.09 (1.01–1.18)	0.03
Urban Jungle	0.90 (0.83–0.99)	0.022	0.91 (0.84–0.99)	0.03
Rural Jungle	1.13 (1.04–1.24)	0.006	1.19 (1.09–1.30)	<0.001
Metropolitan Lima and Callao	0.99 (0.92–1.07)	0.83	1.04 (0.96–1.12)	0.35
**Poverty status**				
Non-poor	Reference		Reference	
Non-extreme poor	0.70 (0.65–0.76)	<0.001	0.72 (0.67–0.78)	<0.001
Extreme poor	0.71 (0.62–0.81)	<0.001	0.68 (0.59–0.78)	<0.001

PR: prevalence ratio; CI: confidence interval; SIS: Comprehensive Health Insurance; EsSalud: Social Health Insurance. aPR: adjusted prevalence ratio. Statistically significant (*p*-value < 0.05). Source: ENAHO 2023.

**Table 4:** Concentration index for formal health service utilization among Peruvian adults, ENAHO 2023.

Socioeconomic Ranking Variable	Outcome	Concentration Index	95% CI	*p*-Value
**Per capita household total expenditure**	Formal health service utilization	0.097	0.083–0.111	<0.001

Note: A positive concentration index indicates that formal health service utilization is concentrated among adults with higher socioeconomic position. The analysis incorporated ENAHO sampling weights. Confidence intervals were estimated using cluster-stratified bootstrap procedures.

**Table 5:** Exploratory stratified analyses of the association between poverty status and formal health service utilization among Peruvian adults, ENAHO 2023.

Stratification Variable	Subgroup	Poverty Status Comparison	N	aPR	95% CI	*p*-Value
**Sex**	Men	Non-extreme poor vs. non-poor	36,949	0.71	0.63–0.80	<0.001
**Sex**	Men	Extreme poor vs. non-poor	36,949	0.66	0.54–0.80	<0.001
**Sex**	Women	Non-extreme poor vs. non-poor	40,701	0.72	0.66–0.79	<0.001
**Sex**	Women	Extreme poor vs. non-poor	40,701	0.69	0.59–0.81	<0.001
**Age group**	18–29 years	Non-extreme poor vs. non-poor	18,220	0.68	0.56–0.83	<0.001
**Age group**	18–29 years	Extreme poor vs. non-poor	18,220	0.70	0.54–0.91	0.008
**Age group**	30–39 years	Non-extreme poor vs. non-poor	14,229	0.74	0.64–0.86	<0.001
**Age group**	30–39 years	Extreme poor vs. non-poor	14,229	0.62	0.49–0.79	<0.001
**Age group**	40–59 years	Non-extreme poor vs. non-poor	26,611	0.72	0.65–0.81	<0.001
**Age group**	40–59 years	Extreme poor vs. non-poor	26,611	0.68	0.53–0.87	0.002
**Age group**	≥60 years	Non-extreme poor vs. non-poor	18,590	0.73	0.64–0.83	<0.001
**Age group**	≥60 years	Extreme poor vs. non-poor	18,590	0.69	0.54–0.87	0.002
**Health insurance status**	Uninsured	Non-extreme poor vs. non-poor	9633	0.42	0.27–0.66	<0.001
**Health insurance status**	Uninsured	Extreme poor vs. non-poor	9633	0.38	0.17–0.85	0.018
**Health insurance status**	Insured	Non-extreme poor vs. non-poor	68,017	0.73	0.68–0.78	<0.001
**Health insurance status**	Insured	Extreme poor vs. non-poor	68,017	0.68	0.60–0.78	<0.001

Note: aPR: adjusted prevalence ratio; CI: confidence interval. Models were stratified by sex, age group, and health insurance status. Within each stratum, poverty status was the main exposure, and models were adjusted for the remaining covariates not used as the stratification variable. Health insurance was categorized as insured versus uninsured to avoid unstable estimates in small insurance categories. Analyses were based on the complete-case sample.

## Data Availability

The ENAHO 2023 dataset analyzed in this study is publicly available and fully anonymized through the microdata repository of the National Institute of Statistics and Informatics of Peru (INEI), accessed on 11 June 2025. The analytic code used to reproduce the results is available from the corresponding author upon reasonable request.

## Abbreviations

The following abbreviations are used in this manuscript:

aPR	Adjusted prevalence ratio
CI	Confidence interval
CIEI-UC	Institutional Research Ethics Committee of Universidad Continental
ENAHO	National Household Survey
EsSalud	Social Health Insurance
INEI	National Institute of Statistics and Informatics
PEN	Peruvian sol
PR	Prevalence ratio
SIS	Comprehensive Health Insurance
Stata	Statistical software developed by StataCorp
USD	United States dollar

## References

[1] Lueckmann S.L., Hoebel J., Roick J., Markert J., Spallek J., von dem Knesebeck O., Richter M. (2021). Socioeconomic Inequalities in Primary-Care and Specialist Physician Visits: A Systematic Review. Int. J. Equity Health.

[2] Kovacs R., Maia Barreto J.O., da Silva E.N., Borghi J., Kristensen S.R., Costa D.R.T., Bezerra Gomes L., Gurgel G.D., Sampaio J., Powell-Jackson T. (2021). Socioeconomic Inequalities in the Quality of Primary Care under Brazil’s National Pay-for-Performance Programme: A Longitudinal Study of Family Health Teams. Lancet Glob. Health.

[3] Alamneh T.S., Teshale A.B., Yeshaw Y., Alem A.Z., Ayalew H.G., Liyew A.M., Tessema Z.T., Tesema G.A., Worku M.G. (2022). Socioeconomic Inequality in Barriers for Accessing Health Care among Married Reproductive Aged Women in Sub-Saharan African Countries: A Decomposition Analysis. BMC Womens Health.

[4] Cai J., Li Y., Li R., Coyte P.C. (2024). Has Socioeconomic Inequality in Perceived Access to Health Services Narrowed among Older Adults in China?. BMC Health Serv. Res..

[5] Blasco-Palau G., Prades-Serrano J., González-Chordá V.M. (2023). Socioeconomic Inequalities as a Cause of Health Inequities in Spain: A Scoping Review. Healthcare.

[6] Lee J.-Y. (2023). Economic Inequality, Social Determinants of Health, and the Right to Social Security. Health Hum. Rights.

[7] Kota K., Chomienne M.-H., Geneau R., Yaya S. (2023). Socio-Economic and Cultural Factors Associated with the Utilization of Maternal Healthcare Services in Togo: A Cross-Sectional Study. Reprod. Health.

[8] Negash H.K., Endale H.T., Aragie H., Tesfaye W., Getnet M., Asefa T., Gela Y.Y., Zegeye A.F. (2025). Barriers to Healthcare Access among Female Youths in Mozambique: A Mixed-Effects and Spatial Analysis Using DHS 2022/23 Data. BMC Public Health.

[9] Turner A.J., Francetic I., Watkinson R., Gillibrand S., Sutton M. (2022). Socioeconomic Inequality in Access to Timely and Appropriate Care in Emergency Departments. J. Health Econ..

[10] Overview of Peru’s Health System: OECD Reviews of Health Systems: Peru 2025. https://www.oecd.org/en/publications/oecd-reviews-of-health-systems-peru-2025_f3ddb6a4-en/full-report/overview-of-peru-s-health-system_10a277e9.html.

[11] Anaya-Montes M., Gravelle H. (2024). Health Insurance System Fragmentation and COVID-19 Mortality: Evidence from Peru. PLoS ONE.

[12] Fagbamigbe A.F., Adeniji F.I.P., Morakinyo O.M. (2022). Factors Contributing to Household Wealth Inequality in Under-Five Deaths in Low and Middle-Income Countries: Decomposition Analysis. BMC Public Health.

[13] Díaz-Ruiz R., Vargas-Fernández R., Rojas-Roque C., Hernández-Vásquez A. (2024). Socioeconomic Inequalities in the Use of Medical Consultation Services in Peru, 2019. Int. J. Equity Health.

[14] Muñoz-del-Carpio-Toia A., Herrera-Añazco P., Benites-Meza J.K., Benites-Zapata V.A. (2025). Antenatal Care Adequacy before and during the COVID-19 Pandemic in Indigenous Populations in Peru. J. Public Health Res..

[15] Andersen R.M. (1995). Revisiting the Behavioral Model and Access to Medical Care: Does It Matter?. J. Health Soc. Behav..

[16] Lederle M., Tempes J., Bitzer E.M. (2021). Application of Andersen’s Behavioural Model of Health Services Use: A Scoping Review with a Focus on Qualitative Health Services Research. BMJ Open.

[17] Marmot M., Friel S., Bell R., Houweling T.A., Taylor S. (2008). Closing the Gap in a Generation: Health Equity through Action on the Social Determinants of Health. Lancet.

[18] World Health Organization Tracking Universal Health Coverage. 2023 Global Monitoring Report. https://www.who.int/publications/i/item/9789240080379.

[19] GBD 2019 Universal Health Coverage Collaborators (2020). Measuring Universal Health Coverage Based on an Index of Effective Coverage of Health Services in 204 Countries and Territories, 1990-2019: A Systematic Analysis for the Global Burden of Disease Study 2019. Lancet.

[20] Pan American Health Organization Expanding Equitable Access to Health Services: Recommendations for Transforming Health Systems toward Universal Health (2022)-PAHO/WHO|Pan American Health Organization. https://www.paho.org/en/documents/expanding-equitable-access-health-services-recommendations-transforming-health-systems.

[21] Oliveira E.C.T., Louvison M.C.P., Duarte Y.A.d.O., de Andrade F.B. (2025). Socioeconomic Inequalities Related to Perceived Difficulty in Accessing Health Services among Older Adults: A Cross-Sectional Analysis of SABE Study Data. PLoS ONE.

[22] Yang J., Zhong Q., Liao Z., Pan C., Fan Q. (2023). Socioeconomic Deprivation, Medical Services Accessibility, and Income-Related Health Inequality among Older Chinese Adults: Evidence from a National Longitudinal Survey from 2011 to 2018. Fam. Pract..

[23] Gordon T., Booysen F., Mbonigaba J. (2020). Socio-Economic Inequalities in the Multiple Dimensions of Access to Healthcare: The Case of South Africa. BMC Public Health.

[24] Houghton N., Bascolo E., Del Riego A. (2020). Socioeconomic Inequalities in Access Barriers to Seeking Health Services in Four Latin American Countries. Rev. Panam. Salud Publica Pan Am. J. Public Health.

[25] McMaughan D.J., Oloruntoba O., Smith M.L. (2020). Socioeconomic Status and Access to Healthcare: Interrelated Drivers for Healthy Aging. Front. Public Health.

[26] Alberto C.K., Pintor J.K., Langellier B., Tabb L.P., Martínez-Donate A.P., Stimpson J.P. (2020). Association of Maternal Characteristics with Latino Youth Health Insurance Disparities in the United States: A Generalized Structural Equation Modeling Approach. BMC Public Health.

[27] Wendt A., Marmitt L.P., Nunes B.P., Dumith S.C., Crochemore-Silva I. (2022). Socioeconomic Inequalities in the Access to Health Services: A Population-Based Study in Southern Brazil. Cienc. Saude Coletiva.

[28] Quizhpe E., Sebastian M.S., Teran E., Pulkki-Brännström A.-M. (2020). Socioeconomic Inequalities in Women’s Access to Health Care: Has Ecuadorian Health Reform Been Successful?. Int. J. Equity Health.

[29] Ministerio de Salud del Perú Política Nacional Multisectorial de Salud al 2030. https://www.gob.pe/institucion/minsa/informes-publicaciones/1127209-politica-nacional-multisectorial-de-salud-al-2030.

[30] Ministerio de Salud del Perú Plan Estratégico Sectorial Multianual PESEM 2024–2030 del Sector Salud. Resolución Ministerial N. 1174-2023-MINSA. https://www.gob.pe/institucion/minsa/normas-legales/4989438-1174-2023-minsa.

